# Society of Physicians and Surgeons

**Published:** 1878-05

**Authors:** Junius M. Hall


### SOCIETY OF PHYSICIANS AND SURGEONS.

Reported by Junius M. Hall, M. D.

(*Regular meeting at Grand Pacific Hotel, Monday evening, April* 8, 1878.)

The President, Dr. Wm. H. Byford, in the chair.

Dr. Wm. G. Dyas, read the second paper of a series of four, upon the lymphatic system. It was devoted exclusively to the subject of alimentation, describing and comparing the manner in which the different organisms assimilate nutriment. Commencing from the lowest grade of organic life as it exists in unicellular animals, then demonstrating the gradual steps nature takes to the development of the typical intestinal canal, he proceeded to show the several objects accomplished by alimentation in the formation and maintenance of the individual, and the continuation of the species; to what extent, moreover, the temperature of the organisms depended on it. In connection with this part of the subject, he showed how different organisms were affected by varying degrees of heat, and how the condition of dryness or moisture influenced the result.

He insisted upon the difficulty there was in giving an adequate definition of what constituted animal life as separate from organic life as seen in vegetables, and was of the opinion that, as yet, logically speaking, we must be content with a mere description of the several attributes of both modes of existence, as observed in their more developed forms.

Dr. Sawyer showed a method of using the forceps during the expulsive stage of labor, in order to prevent extension of the head, thus obviating to some extent the danger of lacerating the perineum.

A letter was read informing the society that the Chicago Medical Society, at its last meeting, adopted the report of the joint committee of the two societies, recommending union and a central place of meeting.

After considerable discussion as to the advisability of disbanding this society, it was decided to defer final action to a future meeting.

---

THE Illinois State Board of Health has decided to issue, as soon as it can be prepared, a directory of all the practitioners in the State, giving their legal status, with other information of importance. This step is prompted by the frequent queries with regard to the standing of practitioners, and is absolutely necessary to give efficiency to the "Act to Regulate the Practice of Medicine." It will contain over 5,000 names. About 4,500 certificates of the different kinds have been issued. Two have been revoked, and no doubt more will be. There are now nearly 300 cases undecided. More have applied than was expected, and the desire has been general to get certificates, even by those who were not compelled by law to do so. In some of the counties, both laws are carried out, owing to the interest taken in them by the profession, and the hearty co-operation of county clerks. As far as can be learned, about 1,100 have left the State or abandoned practice.

THE Third Annual Session of the State Medical Society, of Arkansas, will be held in Fort Smith, on Wednesday, May 1st, 1878. This organization already numbers over two hundred and thirty members, engaged in the practce of medicine in the State. All graduates of medical colleges and universities in good repute with the American Medical Association, interested in the progress of Medical Science, are invited to attend and join the organization.

